# Hypothermic Machine Preservation of the Liver: State of the Art

**DOI:** 10.1007/s40472-018-0183-z

**Published:** 2018-01-22

**Authors:** Andrea Schlegel, Xavier Muller, Philipp Dutkowski

**Affiliations:** 1 The Liver Unit, Queen Elizabeth University Hospital Birmingham, Birmingham, UK; 2 NIHR Liver Biomedical Research Unit, University Hospitals Birmingham, Birmingham, UK; 3 Department of Surgery & Transplantation, Swiss HPB and Transplant Center, University Hospital Zurich, Raemistrasse 100, CH-8091 Zurich, Switzerland

**Keywords:** Machine perfusion, Mitochondria, Hypothermic oxygenated perfusion (HOPE), Metabolic liver function

## Abstract

**Purpose of Review:**

In this review, we highlight which livers may benefit from additional treatment before implantation and describe the concept of hypothermic machine liver perfusion. Furthermore, we explain why cold oxygenated perfusion concepts could potentially lead to a breakthrough in this challenging field of transplantation. Accordingly, we summarize recent clinical applications of different hypothermic perfusion approaches.

**Recent Findings:**

The impact of end-ischemic, hypothermic liver perfusion in liver transplantation is currently assessed by two multicenter, randomized controlled trials. Recently, new applications of hypothermic perfusion showed promising results and recipients were protected from severe intrahepatic biliary complications, despite the use of very extended criteria grafts including donation after circulatory death livers.

**Summary:**

Hypothermic machine liver perfusion is beneficial for high-risk livers and protects recipients from most feared complications. Importantly, such easy approach is currently implemented in several European centers and new markers obtained from perfusate may improve the prediction of liver function in the future.

## Introduction

The worldwide need of organs for transplantation has triggered a revival of machine perfusion techniques, with the aim to rescue organs previously not considered for transplantation. However, based on the excellent results in conventionally stored non-injured liver grafts [1^••^], the aim for machine perfusion in the field appears ambitious; machine perfusion should offer a real repair of high-risk organs before implantation, should also allow testing of organ function, and potentially enable prolonged preservation, if needed for logistic reasons [2]. At the same time, machine perfusion procedures need to be most practical and also affordable. To meet such challenges, an extensive understanding of the underlying mechanism of liver injury and protection is of utmost importance.

This review focuses therefore on recent developments and research in cold perfusion techniques in liver transplantation. First, we discuss which liver grafts would benefit from perfusion approaches. Next, we highlight pros and cons of cold liver perfusion. Third, we report on new findings regarding mechanism of injury and protection during and after cold liver perfusion. Finally, we provide a clinical outlook and report on current human applications.

## Which Livers May Benefit from New Preservation Techniques?

Before the era of cold storage, perfusion of organs prior to transplantation had already received major interest, as the idea behind was to maintain organ function outside of the human body by supplying oxygen and nutrients [3]. However, cooling with modern preservation solutions offered a very simple and cheap way of keeping an organ transplantable for several hours without severe loss of viability [4]. Nowadays, the limits of static preservations techniques have been recognized and machine perfusion techniques receive significant re-interest for their potential advantages in supporting organ function during preservation [5]. Despite this, cold storage remains an easy and successful preservation technique for normal or ideal liver grafts, actually depicted in results of a recent benchmark study in transplantation of cold stored livers [1^••^]. Of note, definitions of extended criteria donor (ECD) livers are somewhat arbitrary and depend on the donation rates and physiology of donors in different countries, e.g., donor age > 60–80 years, hepatic steatosis > 15–30%, cold storage > 10–12 h [6–11]. Many European centers nowadays routinely face liver offers from donors above 60 years of age, together with significant amount of steatosis in the era of NASH [12, 13]. Based on this, the “normal” liver graft today is often already aged between 60 and 70 years, with significant macrosteatosis up to 15% and cold ischemia up to 10 h [8, 13–15]. Such data however differ from the USA, underlined by a significantly higher donor risk index (DRI) in Europe [10]. Extended criteria in Europe therefore include livers with a high amount of macrosteatosis (> 30 or > 40%), prolonged cold ischemia (> 12 h), additional donor warm ischemia (DCD), or a very high donor age (> 80 years) (EASL guidelines [10, 14, 16]). Those liver grafts will likewise need optimization before implantation, especially when combined with risky recipients (re-transplantation, high model of end-stage liver disease—MELD score) [17, 18]. Based on the reported literature, liver grafts have been categorized in Table 1 and potential applications of machine perfusion in extended DBD and DCD livers are described.

**Table 1:** Transmitted risk in DBD and DCD liver transplantation and suggested preservation method

Risk class	Risk parameter	Suggested preservation
Normal graft = ECD graft	-Donor age up to 80 years	Standard cold storage
	-Cold ischemia up to 10 h	
	-Macrosteatosis up to 20%	
Extended ECD	-Donor age > 80 years	Machine perfusion recommended
	-Cold ischemia > 10–15 h	
	-Macrosteatosis > 20%	
“Normal” DCD graft	-Donor age up to 60 years	Standard cold storage
	-Functional donor warm ischemia up to 20 min	
	-Cold ischemia up to 6 h	
	-Macrosteatosis up to 5%	
Extended DCD graft	-Donor age > 60–80 years	Machine perfusion recommended
	-Functional donor warm ischemia > 20 min	
	-Cold ischemia > 6–8 h	
	-Macrosteatosis > 5–20%	
Overextended DCD graft (“high Risk”)^11^	-Donor age > 80 years	Not without machine perfusion
	-Functional donor warm ischemia > 30 min	
	-Cold ischemia > 8 h	
	-Macrosteatosis > 20%	

Combination of > 2 of the risk factors in each risk parameter box

*ECD* extended criteria graft, *DED* donation after brain death, *DCD* donation after circulatory death

## Why Cold Perfusion?

The advantages and disadvantages of hypothermia are both caused by decreased cellular metabolism rates due to slowing down of enzymatic processes of multiple proteins in the cold. Protective effects of hypothermia have been repeatedly recognized in the past centuries since the time of Hippocrates. For example, Napoleon’s battlefield surgeon, Baron Larrey, observed improved survival of injured soldiers left in the snow compared with those treated with warm blankets and hot drinks [19, 20]. However, cold storage of organs without active supply of oxygen and nutrients is limited to the energetic reserves of liver grafts, which are depleted between 24 and 48 h of storage, as anaerobic glycolysis is the main metabolic pathway [21]. This leads slowly to intracellular acidosis, nucleotide depletion, and accumulation of purine metabolites, e.g., hypoxanthine [22, 23]. The time an organ can sustain these conditions depends therefore on cooling to reduce metabolic activity and oxygen requirements and on the use of fluids designed to preserve the intracellular milieu in the absence of proper Na+/K+ pump function [24]. In addition, metabolic function is more difficult to assess at temperatures below 15 °C, especially for livers, where no active bile production is measurable in the cold [25].

In contrast, the concept of full physiological support of organs outside of the body under normothermic conditions aims to avoid metabolic stress, provides oxygen and energy by driving aerobic pathways, and allows testing of organ function [26–28]. Applying however normothermic perfusion after ischemia, e.g., normothermic regional perfusion (NRP) or normothermic ex vivo perfusion, bears also a risk of severe injury.

**Fig. 1:**
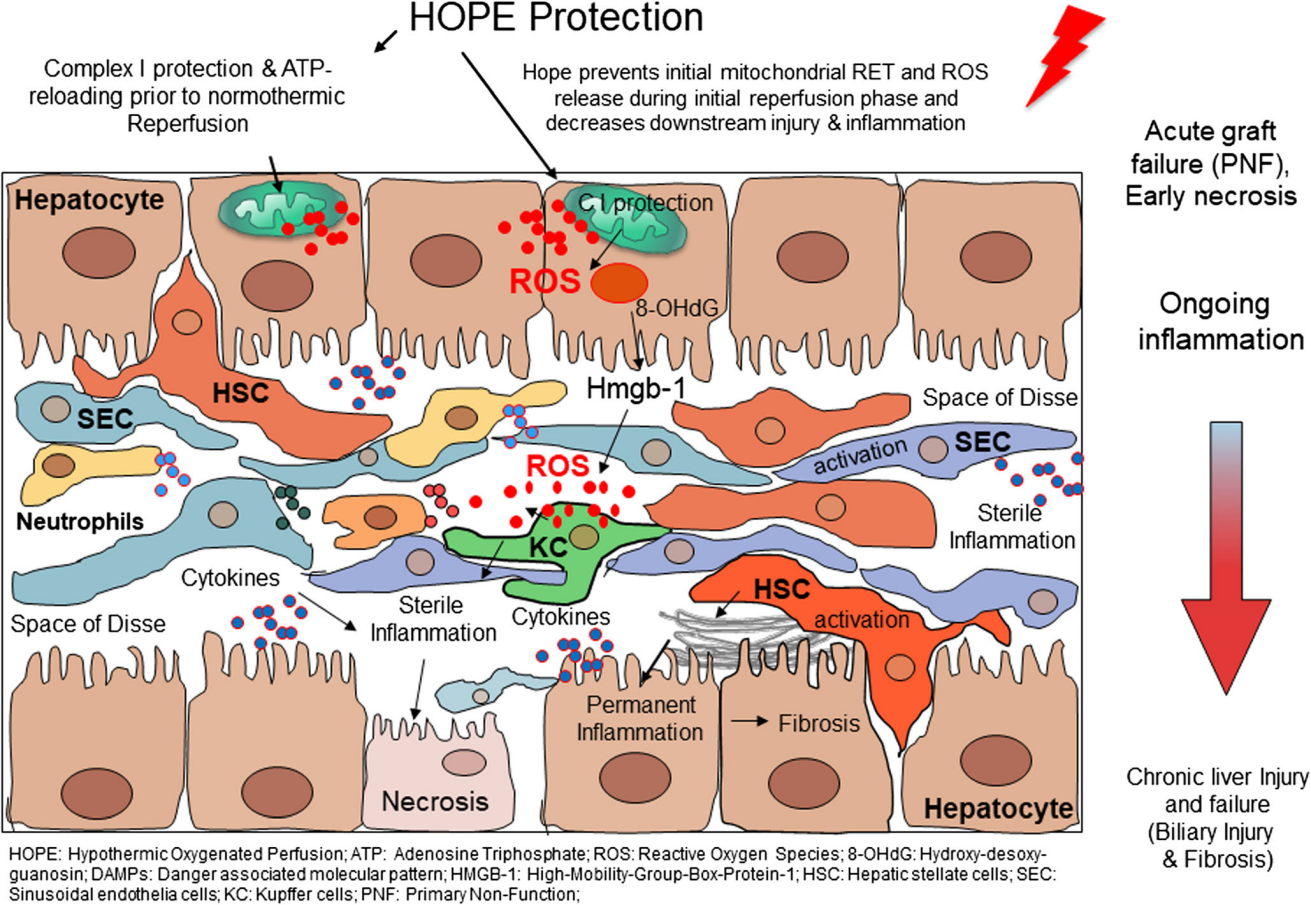
Mechanism of injury following ischemia/reperfusion and protection through hypothermic machine perfusion approaches

Ischemia reperfusion injury occurs when blood supply to a tissue is blocked for minutes to hours and afterwards restored [29]. The current consensus is that a period of ischemia primes the tissue for subsequent damage upon reperfusion [30]. While ischemic cells will die if blood flow is not reestablished, significant damage is initiated during reperfusion [31••]. Thus, paradoxically, the essential therapeutic intervention to treat ischemia, i.e., reperfusion, drives also tissue pathophysiology [32••]. Of note, the first minutes of reperfusion are most critical, as the first damaging and irreversible event is a burst of reactive oxygen species (ROS) produced by mitochondria [33••, 34]. Mitochondrial ROS initiate disrupting of adenosine triphosphate (ATP) production, opening of the mitochondrial permeability transition (MPT) pore, and releasing of danger-associated molecular patterns (DAMPs) [32••, 33••, 34], which lead to sterile inflammation (Fig. 1) [35•], besides activation of the innate immune system [36]. In the long term, such events trigger the formation of fibrotic scar tissue replacing dead cells [37]. The exact causes of mitochondrial ROS upon reperfusion have been controversially discussed [38, 39], but recent studies support the view that complex I is the main site of mitochondrial superoxide production [33••]. Metabolic transitions during ischemia shift electrons to succinate, which acts as an electron store in the absence of oxygen [31••]. Upon reperfusion, succinate fuels reverse electron transfer (RET) between complex II and I, due to high proton motive forces in the first minutes following ischemia [40]. Therapeutic interventions should therefore address accumulation of succinate during ischemia, or its oxidation during reperfusion. For example, inhibition of RET by temporarily blocking of complex II [34] or decreasing mitochondrial ROS by mitochondrial antioxidants is currently explored [41]. In this context, the supply of oxygen to ischemic mitochondria under cold conditions by hypothermic oxygenated organ perfusion is a new and interesting approach, as it addresses several key points:

In contrast to normothermic reperfusion, oxygenation of cells in the cold lead to a very limited ROS release, probably due to a low proton motive force at temperatures below 15 °C (own data). Of note, reverse electron flow is mostly abundant in spite of accumulated succinate (own data). At the same time, forward metabolism of accumulated succinate leads to ATP resynthesis [34, 42]. The phenomenon of shutting down most fueling processes and supporting mitochondrial or chloroplast activity is probably related to a common ancestral process in animals, humans, and plants, enabling cells to survive in winter time by hibernation or winter rest [43, 44]. Reperfusion of ischemic livers, treated by cold oxygenated perfusion, triggers therefore significant less oxidative damage in mitochondria with subsequent less downstream inflammation (Fig. 1) [45, 46]. Importantly, mitochondrial switch from ischemic to fully ATP loaded status needs 1–2 h of cold oxygenated perfusion, which can be performed after cold storage in transplant centers [47]. Such end-ischemic treatment of livers is attractive and cheap, as it needs no additional theater capacity [48].

The disadvantage of this approach is the current lack of methods in testing the energetic status in perfused livers. It is also unclear how long cold oxygenated perfusion could be safely maintained [49••, 50]. Upcoming research, however, is awaited and analysis of perfusate during cold oxygenated perfusion by NMR techniques will likewise allow measuring metabolic function of livers during cold perfusion.

Importantly, the effectiveness of an end-ischemic cold liver oxygenation has been further paralleled by numerous studies from the group of Minor who performed oxygen persufflation in the livers and kidneys under hypothermic conditions [51]. Likewise, normothermic oxygenated short-term perfusion provides also protection of the kidneys in spite of significant cold storage periods before end-ischemic perfusion [52, 53].

## Different Technical Aspects of Hypothermic Liver Perfusion

The majority of experimental studies published on hypothermic machine perfusion (HMP) in the last 20 years involved mainly ex vivo liver perfusions without implantation [54]. In accordance with experiences from the kidney perfusions, HMP in livers was initially applied continuously and demonstrated improved hepatocyte and endothelial cell viability compared to simple cold storage [55–57]. The experimental conditions used, however, varied largely, and most of these studies were performed on liver grafts with no or only minor injury.

Regarding the perfusion route, two different approaches are competitively used. Single portal vein perfusion was usually preferred in rat livers [57–62], while seven studies explored liver integrity using dual perfusion techniques via hepatic artery and portal vein in pig livers [55, 56, 63–67]. Although dual perfusion through the hepatic artery and the portal vein failed to show clear advantages in ex vivo models [55, 64, 66, 68], advocates of this dual technique repeatedly emphasize better supply of oxygen to the peribiliary vascular plexus [69–71]. Most of the interlobular biliary branches are however also reached by portal branches, and the debate is ongoing regarding the amount of oxygen needed in the portal vein during HMP. In discarded human livers, Jomaa et al. showed feasibility of short and end-ischemic machine liver perfusion at 4–8 °C using dual vs single portal vein or hepatic artery [72]. No histologically difference appeared in these livers comparing different perfusion routes [72]. The technique of dual hypothermic machine liver perfusion has first been transferred into clinical practice by the group of James Guarrera, who reported outcomes of 20 human livers transplanted after dual HMP [73].

Next to the perfusion route, the temperature during HMP has been described at a wide range between 1 and 22 °C [74]. Metabolic activity appears more depressed at lower temperature, while the perfusate viscosity increases [75]. In this context, higher vascular resistances have been reported during HMP, which transmit an increased risk of endothelial injury in the liver sinusoids, which becomes more evident when cold perfusion is prolonged and high perfusion pressures are used [60, 76, 77]. Ex situ perfusion experiments are therefore limited to relatively short intervals between 2 and 24 h [74]. In addition, high perfusion pressures were shown to induce endothelial and Kupffer cell injury and most professionals therefore perform HMP at low portal vein pressure of 3–5 mmHg and low arterial pressure of 20–30 mmHg [78].

Tissue oxygenation during HMP simply relies on the dissolved oxygen in a blood-free perfusate [79], and the amount of oxygen needed in the perfusate is another matter of debate. Experimental studies have described a wide range of perfusate oxygenation between 10 and 106 kPA [74, 80]. Importantly, such cold oxygenation enables graft mitochondria to sufficiently produce and restore cellular energy, which increases significantly already within the first hour of perfusion [81–83]. Based on this, majority of centers, who apply HMP in liver transplantation, use it only for a short end-ischemic period [78, 84, 85]. Another advantage of HMP is the much easier technical approach because machine transport is not necessary.

Multiple perfusion solutions including Belzer UW solution and Vasosol with different variations were assessed in experimental studies [5, 74]. For example, low potassium concentrations were found to be protective and experimental studies showed a decreased vascular resistance of livers during cold perfusion [60]. Although several additives, i.e., reactive oxygen scavengers, vasodilators, and amino acids, have been assessed in experiments to improve perfusion quality of livers, none of these substances are routinely used in clinical practice yet [73].The majority of perfusion experiments has however been performed with Belzer UW machine perfusion solution [86]. Importantly, this UW perfusion solution is used for most liver perfusions today, despite the fact that this solution has initially been developed to perfuse kidneys and achieved CE certificate for this application.

## Clinical Applications

Hypothermic dual (portal vein and hepatic artery) perfusion of twenty standard DBD human livers was first reported in 2010 by Guarerra et al. [73]. Machine perfusion was applied after previous 8–9 h cold storage and transport of organs to the recipient center. Prior to implantation, livers underwent 3–7 h HMP with relatively high flow rates of 0.667 ml/g liver weight/min. Importantly, no additional oxygen was supplemented into the perfusate and pO_2_ levels in perfusates ranged between 120 and 160 mmHg [73]. The team of James Guarrera performs the end-ischemic dual cold liver perfusion (cannulating portal vein and hepatic artery) using the modified Medtronic PBS device® (Medtronic Minneapolis). A Vasosol®-based perfusate, supplemented with vasodilators and antioxidants, is continuously circulated through the liver at temperatures between 4 and 8 °C [73]. Of note, the perfusion flows are adjusted to the liver weight (0.667 ml perfusate/g liver/min) and perfusion pressures are monitored [73]. Perfusion resulted in significantly less peak enzyme release and shorter hospital stay, as well as less early allograft dysfunction (EAD) compared to a non-randomized control group. In a further report, the same investigators recently showed less biliary complications after hypothermic perfusion to marginal DBD organs (Table 2) [85].

**Table 2:** Clinical studies involving hypothermic (HMP) and hypothermic oxygenated perfusion (HOPE), prior to liver transplantation between 2014 and 2017

Author	Year	Model	Species	*n*	Temp (°C)	Perfusion duration (h)	Perfusion route	OLT	Endpoints	Outcome
Schlegel et al. [56]	2017	DCD	Human	50	10	2	PV	Yes	Post-reperfusion syndrome, graft function, rate of PNF, HAT and ischemic cholangiopathy, graft survival	HOPE-treated extended DOD liver grafts showed significant improved 5-year graft survival due to less PNF, HAT, and ischemic cholangiopathy. Control group = untreated DCD, matched according to cold storage
Van Rijn et al. [55]	2017	DCD	Human	10	10	2	PV + HA	Yes	Liver function, ATP content, graft and patient survival	D-HOPE treatment restored hepatic ATP, protect from reperfusion injury and improved 6 and 12 month graft survival
De Carlis et al. [65]	2017	DBD	Human	2	10	6–8	PV + HA	Yes	General outcomes, graft function, complications, survival	Good initial graft function and no complications in the first 5 months. No control group
De Carli s et al. [49^••^]	2017	DCD	Human	1	10	3	PV + HA	Yes	Graft function, general outcomes and biliary complication	No biliary complications in the first 5 months. No control group
Dutkowski et al. [53]	2015	DCD	Human	25	10	1–2	PV	Yes	Transfusion, early allograft dysfunction, graft survival	HOPE-treated extended DOD liver grafts showed comparable good outcomes to matched low risk primary DEB grafts
Guarrera et al. [52]	2015	ECD	Human	20	4–8	4–7	PV + HA	Yes	Incidence of PNF, EAD, vascular complication 1-year graft and patient, survival, incidence of biliary complication	HMP significantly decreased, EAD, hospital stay and showed significantly less biliary complications
Dutkowski et al. [48]	2014	DCD	Human	8	10	1–2	PV	Yes	Liver function, cholestasis parameter, costs, ICU and hospital stay, biliary complications, graft survival	HOPE-treated DOD liver grafts showed significant improved survival and less ischemic cholangiopathy

DOD Maastricht II category; DOD Maastricht Ill category; Perfusion device: All groups used the liver assist device, apart from J. Guarrera, who applies the HMP through a non-pulsatile pump (Medtronic, Minneapolis, MN)

*HMP* hypothermic machine perfusion, *HOPE* hypothermic oxygenated perfusion, *ECD* extended criteria donor; *HID* donation after brain death, *DOD* donation after circulatory death, *PV* portal vein, *HA* hepatic artery, *ICU* intensive care unit, *EAD* early allograft dysfunction, *PNF* primary non-function, *HAT* hepatic artery thrombosis

**Fig. 2:**
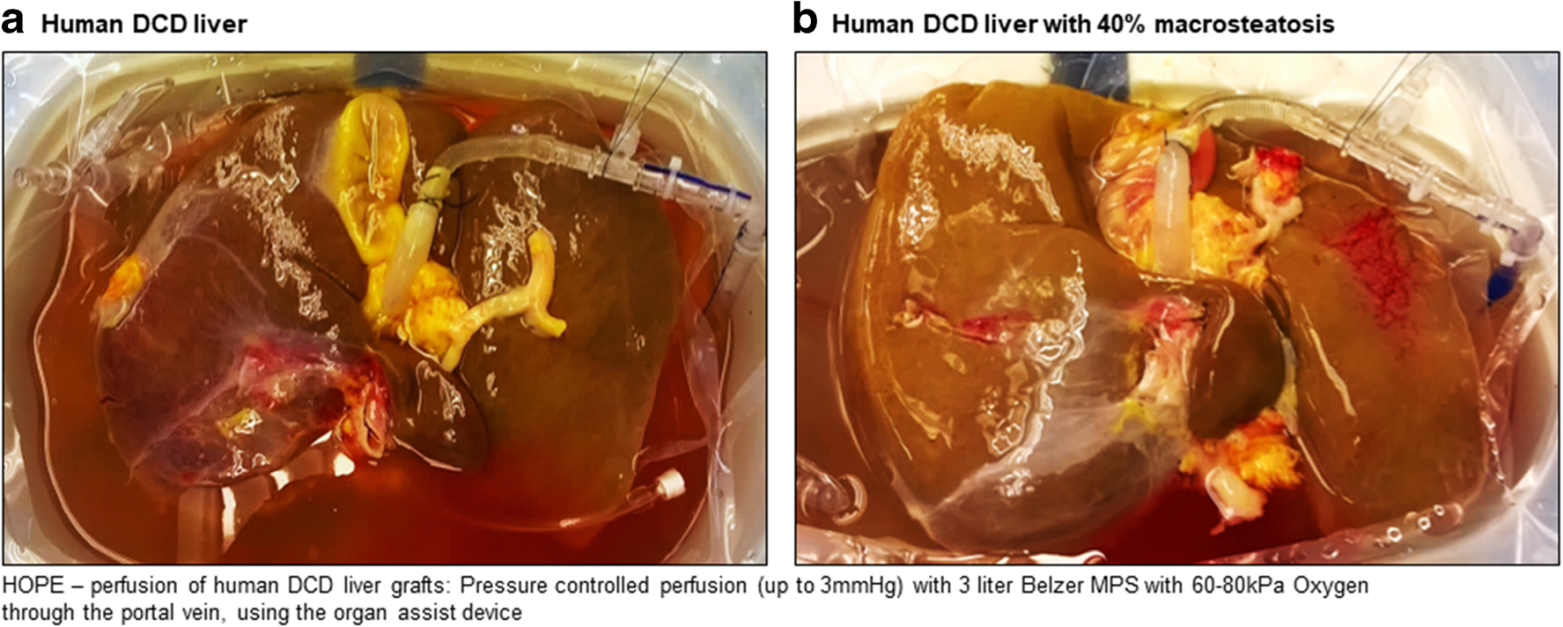
Examples of hypothermic oxygenated perfusion of liver grafts prior to implantation

Consistent to these results, hypothermic oxygenation perfusion (HOPE) has been shown to be effective in human DCD liver grafts, with less occurrence of intrahepatic biliary complications as compared to matched un-perfused DCD livers. Our practice of HOPE is based on more than 15 years of experimental research in several small and large animal transplant models [82, 87–89], as well as on human practice in DCD livers [48, 84]. The perfusion is performed solely through the portal vein, in an open system (Liver Assist device (Organ Assist®)), where the liver swims in the cold perfusion solution and the perfusate flows out of the vena cava passively, to avoid sinusoidal congestion together with adjusting the maximum perfusion pressure at 3 mmHg (Fig. 2) [82]. Under these conditions, the perfusion flow ranges between 150 and 250 ml/min [82, 90]. We perfuse with 3 l of recirculating Belzer machine perfusion solution (MPS) at temperatures between 8 and 12 °C and a high oxygen saturation (60–80 kPa) [2]. Perfusion is maintained for at least 1 h, but is generally performed during recipient hepatectomy until graft implantation without need of an extra theater setting.

Most importantly, HOPE treatment appears sufficient by single portal vein perfusion, as the entire intra- and extrahepatic biliary system is positively effected through multiple collaterals between portal vein and hepatic artery [48, 84, 91]. Furthermore, at hypothermic temperatures, single portal vein perfusion has been demonstrated to sufficiently protect the biliary tree, in spite of long donor warm ischemia times. The group from Groningen has reported the first ten extended DCD liver grafts, transplanted after dual HOPE (D-HOPE), where hypothermic oxygenated perfusion was applied through both hepatic artery and portal vein with subsequent transplantation. Importantly, no graft loss was described in the D-HOPE group compared to un-perfused controls (Table 2) [78]. The D-HOPE technique equals the HOPE technique and the team of R. Porte perfuses livers with the same liver assist device, using 4 l of Belzer UW solution supplemented with glutathione and oxygen at partial pressures of at least 450 mmHg [78]. End-ischemic cold perfusion in Groningen is performed at 10° for at least 2 h, and cannulation of the hepatic artery is achieved using a large supra-truncural aortic patch provided by the retrieval surgeon in order to not directly cannulate the hepatic artery to prevent arterial injury [78].

Randomized trials have been initiated to further evaluate the effect of HOPE on DBD and DCD liver grafts (hope-liver.com—Zurich, Groningen Institute for Organ transplantation—GIOT: University Hospital RWTH Aachen, Aachen, Germany). In the first long-term outcome analysis after HOPE, 50 recipients of extended DCD livers experienced a similar 5-year graft survival after HOPE treatment, when compared to lowest risk, primary DBD liver transplantations [92]. HOPE has also been recently applied in Maastricht type II DCD livers following standard NRP and cold storage in Italy [49^••^] and the perfusion protected DCD kidneys, as demonstrated recently in a rodent model of kidney transplantation [93, 94].

## Conclusions and Future Perspective

Improvement of the quality of liver grafts and prediction of organ function before implantation are the two main issues to allow the safe use of injured organs. Most efforts should therefore be directed to further develop dynamic preservation methods, which will likewise replace static cold storage in high-risk grafts. In this context, thresholds need to be defined, and machine perfusion techniques should be compared. For example, the impact of new perfusion devices, e.g., the Transmedics® machine for normothermic perfusion, is awaited [95], and potential new oxygen carriers, e.g., Hemopure® replacing RBCs during sub- and normothermic perfusion, are under investigation [96]. Importantly, modern analytical technologies (e.g., proteomics, metabolomics) are currently applied on liver tissue and perfusate and may help to explore new biomarkers, which are urgently needed to assess graft quality and predict not only the necessary perfusion duration but also liver function after subsequent transplantation.

## Abbreviations

ATP: Adenosine triphosphate
DAMPs: Danger-associated molecular patterns
DBD: Donation after brain death
DCD: Donation after circulatory death
D-HOPE: Dual hypothermic oxygenated perfusion
DWIT: Donor warm ischemia time
EAD: Early allograft dysfunction
EASL: The European Association for Study of the Liver
ECD: Extended criteria donor
HMP: Hypothermic machine perfusion
HOPE: Hypothermic oxygenated perfusion
IC: Ischemic cholangiopathy
KCs: Kupffer cells
MELD: Model of end-stage liver disease
MPT pore: Mitochondria permeability transition pore
ROS: Reactive oxygen species
SEC: Sinusoidal endothelial cells
TLR-4: Toll-like-receptor-4
