# Correction to: Hypothermic Machine Preservation of the Liver: State of the Art

**DOI:** 10.1007/s40472-018-0187-8

**Published:** 2018-03-03

**Authors:** Andrea Schlegel, Xavier Muller, Philipp Dutkowski

**Affiliations:** 1 The Liver Unit, Queen Elizabeth University Hospital Birmingham, Birmingham, UK; 2 NIHR Liver Biomedical Research Unit, University Hospitals Birmingham, Birmingham, UK; 3 Department of Surgery & Transplantation, Swiss HPB and Transplant Center, University Hospital Zurich, Raemistrasse 100, 8091 Zurich, Switzerland

**Correction to: Curr Transplant Rep (2018)**

10.1007/s40472-018-0183-z

The article Hypothermic Machine Preservation of the Liver: State of the Art, written by Andrea Schlegel, Xavier Muller and Philipp Dutkowski, was originally published electronically on the publisher’s internet portal (currently SpringerLink) on January 22, 2018, without open access.

With the author(s)’ decision to opt for Open Choice the copyright of the article changed on February 2018 to © The Author(s) 2018 and the article is forthwith distributed under the terms of the Creative Commons Attribution 4.0 International License (http://creativecommons.org/licenses/by/4.0/), which permits use, duplication, adaptation, distribution, and reproduction in any medium or format, as long as you give appropriate credit to the original author(s) and the source, provide a link to the Creative Commons license, and indicate if changes were made.

The original article has been corrected.

